# Deficiency of UCHL1 results in insufficient decidualization accompanied by impaired dNK modulation and eventually miscarriage

**DOI:** 10.1186/s12967-024-05253-0

**Published:** 2024-05-20

**Authors:** Jie Zhang, Mingxing Xue, Jiefang Huang, Shan He, Lingqiao Zhu, Xiaonan Zhao, Bei Wang, Tingwang Jiang, Yanyun Zhang, Changhong Miao, Guoqiang Zhou

**Affiliations:** 1 Department of Gastrointestinal Surgery, The Affiliated Changshu Hospital of Nantong University, 68 South Haiyu Road, Changshu, 215500 China; 2 Department of Anesthesiology, Zhongshan Hospital, Fudan University, 180 Fenglin Road, Shanghai, 200032 China; 3 Institutes for Translational Medicine, Children’s Hospital of Soochow University, Soochow University, Suzhou, China; 4 Shanghai Institute of Nutrition and Health, Chinese Academy of Sciences, Shanghai, China; 5 Gusu College, Nanjing Medical University, Nanjing, China

**Keywords:** UCHL1, Miscarriage, Decidualization, dNK modulation

## Abstract

**Background:**

Miscarriage is a frustrating complication of pregnancy that is common among women of reproductive age. Insufficient decidualization which not only impairs embryo implantation but disturbs fetomaternal immune-tolerance, has been widely regarded as a major cause of miscarriage; however, the underlying mechanisms resulting in decidual impairment are largely unknown.

**Methods:**

With informed consent, decidual tissue from patients with spontaneous abortion or normal pregnant women was collected to detect the expression profile of UCHL1. Human endometrial stromal cells (HESCs) were used to explore the roles of UCHL1 in decidualization and dNK modulation, as well as the mechanisms involved. C57/BL6 female mice (7–10 weeks old) were used to construct pregnancy model or artificially induced decidualization model to evaluate the effect of UCHL1 on mice decidualization and pregnancy outcome.

**Results:**

The Ubiquitin C-terminal hydrolase L1 (UCHL1), as a deubiquitinating enzyme, was significantly downregulated in decidua from patients with miscarriage, along with impaired decidualization and decreased dNKs. Blockage of UCHL1 led to insufficient decidualization and resultant decreased expression of cytokines CXCL12, IL-15, TGF-β which were critical for generation of decidual NK cells (dNKs), whereas UCHL1 overexpression enhanced decidualization accompanied by increase in dNKs. Mechanistically, the promotion of UCHL1 on decidualization was dependent on its deubiquitinating activity, and intervention of UCHL1 inhibited the activation of JAK2/STAT3 signaling pathway, resulting in aberrant decidualization and decreased production of cytokines associated with dNKs modulation. Furthermore, we found that inhibition of UCHL1 also disrupted the decidualization in mice and eventually caused adverse pregnancy outcome.

**Conclusions:**

UCHL1 plays significant roles in decidualization and dNKs modulation during pregnancy in both humans and mice. Its deficiency indicates a poor pregnancy outcome due to defective decidualization, making UCHL1 a potential target for the diagnosis and treatment of miscarriage.

**Graphical Abstract:**

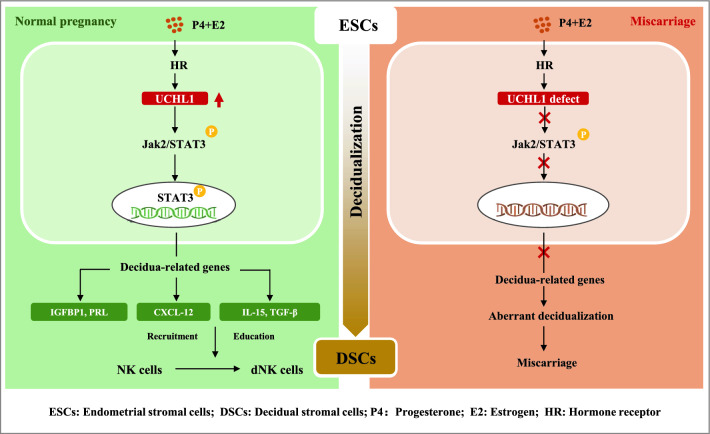

**Supplementary Information:**

The online version contains supplementary material available at 10.1186/s12967-024-05253-0.

## Background

Miscarriage, also known as spontaneous abortion, is a distressing reproductive complication during early pregnancy that affects 15.3% of clinically recognized pregnancies [1], and it can be caused by various factors, such as chromosomal errors, uterine infections, and endocrine abnormalities [2]. Decidualization defect is one of the major maternal causes of miscarriage [3]; however, the underlying molecular mechanisms regulating decidualization and the pathology of miscarriage related to impaired decidualization remain largely unknown.

Decidualization refers to the transformation of endometrial stromal cells (ESCs) into specialized secretory decidual stromal cells (DSCs), which provide a ‘fertile ground’ for embryo implantation [4, 5]. Moreover, the decidualized endometrium plays crucial roles in protecting the embryo from maternal immune rejection by communicating with decidual immune cells (DICs) to establish an immune-tolerant niche at the maternal–fetal interface [6]. Decidual NK cells (dNKs) are the predominant immune population and account for approximately 70% of all DICs in early-pregnancy decidua [6], and their recruitment and education are largely dependent on chemokines and cytokines secreted by DSCs [7–9]. Hence, defective decidualization not only impedes embryo implantation but also disrupts the development of fetomaternal immunotolerance, ultimately leading to miscarriage. Accordingly, the molecular mechanism underlying aberrant decidualization is an urgent issue to be explored.

Ubiquitination and deubiquitination are dynamic, multifaceted post-translational modifications that play a dominant role in protein function and degradation [10]. This modification, orchestrated by numerous ubiquitin ligases or deubiquitinating enzymes, controls almost all fundamental biological processes of metazoans, including cell division, differentiation and migration [11]. Increasing evidences suggest that ubiquitinating and deubiquitinating modifications are involved in pregnancy. For example, loss of the deubiquitinating enzyme USP36 causes preimplantation embryonic lethality in mice by modulating the stability of DHX33 [12]. Cullin 1 (CUL1), a scaffold protein in cullin-based ubiquitin ligases, promotes trophoblast invasion and migration [13]. Moreover, pregnancy complications, especially miscarriage, have been reported to be related to dysregulated ubiquitination [14–16]. However, it is unclear whether the processes of ubiquitination and deubiquitination modulate endometrial decidualization.

Ubiquitin C-terminal hydrolase L1 (UCHL1) is a deubiquitinating enzyme that was first studied in the nervous system and is associated with synaptic function and contextual memory [17]. Aberrant function of UCHL1 has been reported to be involved in neurodegenerative disorders such as Parkinson’s disease [18] and Alzheimer’s disease [19]. Moreover, UCHL1 has been reported as a multifunctional deubiquitinating enzyme involved in cancer invasion, metastasis [20] and chemoresistance [21]. Our previous study revealed that UCHL1 negatively regulated the immunosuppressive activity of mesenchymal stem cells [22]. Additionally, within the mammalian reproductive system, UCHL1 plays an essential regulatory role in controlling mammalian oocyte development and spermatogenesis [23]. A recent finding reveals that within the uterus during early pregnancy of mice, UCHL1 is specifically expressed in decidual cells [24]; however, the roles of UCHL1 have not been explored in miscarriage related to impaired decidualization.

In the present study, we found that the expression of UCHL1 was dramatically decreased in decidua from patients suffering miscarriage, accompanied by aberrant decidualization indicated by the downregulation of decidual markers IGFBP1 and PRL [25] and decreased dNKs due to reduced production of CXCL12, IL15 and TGF-β from DSCs. Blocking UCHL1 inhibited endometrial decidualization and resulted in decreased percentage of dNKs. Conversely, UCHL1 overexpression enhanced the decidualization of ESCs and then promoted the recruitment and education of dNKs, which was dependent on its deubiquitinating activity. Activation of the JAK2/STAT3 signaling pathway, which is essential for decidualization [26], was inhibited when UCHL1 was treated with its specific inhibitor or shRNA but was enhanced in UCHL1-overexpressing ESCs. Furthermore, the enhanced decidualization and cytokine expression induced by UCHL1 overexpression were reversed by STAT3 inhibition. These results indicated that the promotion of decidualization by UCHL1 was dependent on the activation of JAK/STAT3 signaling. Moreover, we verified that UCHL1 played an important role in maintaining successful decidualization and pregnancy in mice, suggesting a conserved function of UCHL1 in pregnancy, which made it feasible to test the efficacy of medicine targeting UCHL1 on miscarriage in a mouse model. Our findings provide evidence that UCHL1 is critical for decidualization as well as the recruitment and education of dNKs and therefore represents a potential target for the diagnosis and treatment of miscarriage.

## Methods

### Human sample collection

This study was approved by the Medical Research Ethics Committee of the Affiliated Changshu Hospital of Nantong University, Changshu, China (ethics approval number: 2018037). Written informed consent was obtained from all subjects. Human endometrial samples and peripheral blood samples were collected from patients with clinically normal pregnancies terminated for nonmedical reasons (n = 20) and unexplained spontaneous abortions (n = 20) at the Affiliated Changshu Hospital of Nantong University, Changshu, China. Criteria for a normal pregnancy include no history of spontaneous abortion or abnormal birth, absence of abdominal pain, vaginal bleeding, fever, or any other evident discomfort after conception. Preoperative examination revealed no pathogen infection and ultrasound examination confirmed the presence of original cardiac tube pulsation without significant uterine effusion. The criteria for unexplained spontaneous abortion include the absence of fetal heart tube pulsation during two consecutive vaginal ultrasound examinations conducted one week apart in early pregnancy. Other potential causes such as trauma, fever, genital malformation, infection, or chromosome abnormality were ruled out. Clinical characteristics of patients with unexplained spontaneous abortions and clinically normal pregnancy are presented in supplementary Table 2.

### Animals

C57BL/6 mice were purchased from the Shanghai Laboratory Animal Center of the Chinese Academy of Sciences (CAS, Shanghai, China). The animals were housed in a specific pathogen-free (SPF) facility at Shanghai Institutes for Biological Sciences, CAS. All animal experiments were conducted following the guidelines of the Institutional Animal Care and Use Committee of Shanghai Institutes for Biological Sciences, CAS (ethics approval number: 20200306-028), and complied with the Guide for the Care and Use of Laboratory Animals published by the U.S. National Institutes of Health.

### Mouse model

Adult C57BL/6 mice (7–10 weeks old) were mated to establish pregnancy. The presence of a vaginal plug was considered day 0 of gestation. To block UCHL1 in-vivo, a working solution was prepared by adding 50 μL of LDN57444 (dissolved in DMSO at 4 mg/mL) or DMSO to 950 μL of corn oil. Subsequently, the mice were injected i.p. daily with the identical volume of working solution containing DMSO (control group) or 0.4 mg/kg LDN57444 (UCHL1 blockade group; S7135, Selleckchem, USA) from gestational day (GD) 5 to 7 (n = 6 per group). The mice were sacrificed on GD8 and the uterine tissues were collected.

The establishment of artificially induced decidualization has been previously described [27]. Briefly, C57BL/6 female mice at 7–8 weeks of age were ovariectomized (n = 6 per group). Two weeks after ovariectomy, the mice were injected with 100 ng of E2 daily for three days. After a two-day interval, the mice received daily injections of 1 mg of P4 and 6.7 ng of E2. Six hours after the third E2 and P4 injection, the right uterine horn was stimulated by 50 µL of sesame oil, while the contralateral horn remained untreated as a control. Daily injection of E2 and P4 was continued for 5 days to induce decidualization of the endometrium. The mice were sacrificed on Day 6 for tissue collection.

### Isolation of DSCs and DICs

Decidual tissues were washed twice with PBS plus penicillin–streptomycin solution (P/S), and DSCs were isolated as soon as possible according to the previously described method [28]. Briefly, decidual tissues were cut into pieces and digested with 0.1% collagenase type IV (Roche, Vienna, Austria) and 0.002% DNase I (Sigma-Aldrich, Darmstadt, Germany) in DMEM/F-12 medium for 30 to 60 min at 37 °C. After digestion, the tissue pieces were filtered through sterile gauze pads (200 mesh) to remove cellular debris. The cell suspension was then centrifuged at 1500 rpm for 8 min, and the supernatant was discarded. Collected cells were resuspended in DMEM/F-12 medium, layered over different concentrations (60%, 40% and 20% from bottom top) of Percoll (GE Healthcare, Chicago, United States) and centrifuged at 2000 rpm for 20 min. The cells from the 20%/40% interface mainly consisted of DSCs, while those from the 40%/60% interface mainly consisted of decidual immunocytes (DICs). Finally, isolated DSCs were cultured in dishes with DMEM/F-12 supplemented with 10% FBS and incubated in a humidified incubator with 5% CO2 at 37 °C. Isolated DICs were cultured in RPMI-1640 medium (HyClone, Utah, United States) containing 10% FBS (Gibco, Massachusetts, United States) and 50 µg/mL penicillin/streptomycin.

### pNK cell isolation

Peripheral blood mononuclear cells (PBMCs) were isolated from blood samples collected from healthy pregnant women in the first trimester by Ficoll-Hypaque (Sigma-Aldrich) density gradient centrifugation at 800×*g* for 20 min. According to the manufacturer’s instructions (MiltenyiBiotec, Bergisch Gladbach, Germany), peripheral NK cells (pNKs) were obtained through negative selection using the human NK cell isolation kit. pNKs were cultured in RPMI-1640 medium (HyClone) supplemented with 10% FBS (Gibco) and 50 µg/mL penicillin/streptomycin. The purity of CD45^+^CD3^−^CD56^+^ pNKs was > 90%, as determined by flow cytometry assays (data not shown).

### Cell coculture assay

For the transwell assay, DSCs isolated from different samples were cultured in medium as described above and treated with LDN57444 or identical volume of DMSO as indicated. The culture medium was then collected in 24-well plates. pNKs (1 * 10^5^ cells/well) were placed in the upper compartment of the Transwell chamber inserts (5 μm aperture, Corning, Painted Post, NY, USA). After 48 h of coculture, the number of dNKs in the lower compartments was counted.

For the coculture assay, isolated DSCs or decidualized HESCs (described below) were seeded in 24-well plates and treated LDN57444 or identical volume of DMSO as control for 24 h. Then, the culture medium was discarded and pNKs were seeded into the plate to coculture with these cells for another 48 h. The suspended cells were collected for flow cytometric analysis.

### Flow cytometry assays

pNKs cocultured with DSCs or decidual HESCs were collected, and the expression of CD45, CD3, CD56 and CD16 was analyzed by flow cytometry assays. Briefly, the collected pNKs were washed twice with PBS and stained with PerCP anti-human CD45 antibody (Biolegend, San Diego, CA, USA), FITC anti-human CD3 antibody (Biolegend), BV421 anti-human CD56 antibody (Biolegend) and APC-Cy7 anti-human CD16 antibody (Biolegend) diluted in PBS with 2% FBS at 4 °C for 30 min. pNKs were then washed twice and analyzed by flow cytometry assays.

DICs isolated from decidual tissue of mice treated with DMSO or LDN57444 were collected, and the expression of CD45, CD3 and NK1.1 was analyzed by flow cytometry assays. The procedure was described above, and the antibodies used were APC-Cy7 anti-mouse CD45 antibody (Biolegend), FITC anti-mouse CD3 antibody (Biolegend) and BV421 anti-mouse NK1.1 antibody (Biolegend).

### Quantitative real-time PCR

Total RNA was extracted from cells or tissues using TRIzol Reagent (Sigma-Aldrich) and subsequently reverse-transcribed into cDNA using the PrimeScript RT Master kit (TaKaRa, Shiga, Japan). Quantitative real-time PCR was performed using FastStart Universal SYBR Green Master Kits (Roche) on a ViiA7 Real-time PCR System (Applied Biosystems) according to the manufacturer’s instructions. The sequences of the primers are listed in Supplementary Table 1.

### Western blot

Total proteins were extracted using lysis buffer (150 mM NaCl, 1% Triton X-100, 1% sodium deoxycholate, 0.1% SDS, 50 mM Tris (pH 7.4), 5 mM EDTA) containing a protease inhibitor cocktail and a phosphatase inhibitor cocktail (Sigma-Aldrich). Then, protein lysates were electrophoresed via SDS‒PAGE and analyzed by western blotting with antibodies against UCHL1 (Abcam CAT# ab8189), IGFBP1 (Cell Signaling Technology, CST CAT# 31025), GAPDH (CST CAT# 2118, RRID: AB_561053), β-actin (CST CAT# 4970, RRID:AB_2223172), P-ERK1/2 (CST CAT# 4370, RRID:AB_2315112), ERK1/2 (CST CAT# 4695, RRID:AB_390779), FOXO1 (CST CAT# 2880, RRID:AB_2106495), JAK2 (CST CAT# 3230), P-STAT3 (CST CAT# 9145), and STAT3 (CST CAT# 9139).

### Immunohistochemistry

Human and mouse tissue samples were fixed in 4% paraformaldehyde, embedded in paraffin after dehydration, cut at 5 µm thickness and mounted on slides. The paraffin slides were then deparaffinized and rehydrated using a graded alcohol series before H&E staining or immunohistochemistry (IHC). For IHC, the slides were boiled in 10 mM sodium citrate buffer (pH 6.0) for 10 min and then naturally cooled to room temperature. After blocking with 5% bovine serum albumin in PBS (pH 7.5), the samples were incubated overnight at 4 °C with anti-UCHL1 (Abcam CAT# ab8189), followed by incubation with an HRP-conjugated secondary antibody. Immunoreactivity was detected using a DAB kit (Gene Tech, South San Francisco, United States).

### Immunofluorescence

Frozen samples of human decidual tissue, embedded in optimum cutting temperature compound (OCT, Sakura Finetek), were sectioned at 7 μm thickness for immunofluorescence. After washing with PBS, the sections were fixed with a mixture of MeOH/acetone (1:1) at − 20 °C for 5 min and blocked with 1% BSA for 1 h at 37 °C. Mouse anti-human UCHL1 (Abcam CAT# ab8189) and rabbit anti-human IGFBP1 (Abcam CAT# ab111203) were applied to slides overnight at 4 °C, followed by incubation with goat anti-mouse Alexa Fluor 488-IgG or goat anti-rabbit Alexa Fluor 555-IgG (eBioscience, California, United States) for 1 h at 37 °C in the dark. Finally, DAPI staining was performed.

### Cell culture and in vitro decidualization

The immortalized human endometrial stromal cell line T-HESCs (ATCC CAT# CRL-4003TM, RRID: CVCL_C464) was a gift from Professor Haibin Wang from the Institute of Zoology, CAS (Beijing, China)^31^. HESCs were cultured in DMEM/F-12 medium containing 10% FBS and 50 µg/mL penicillin/streptomycin with 1 mM sodium pyruvate, 1% insulin-transferrin-selenium (ITS), 3.1 g/L glucose, 1.5 g/L sodium bicarbonate and 500 ng/mL puromycin (all from Thermo Fisher Scientific). The culture medium was replenished every three days. In vitro decidualization of HESCs was induced as previously reported^3^. In brief, HESCs were treated with DMEM/F-12 containing 2% FBS, 10 µM medroxy progesterone (MPA, Selleck Chemicals, Texas, United States) and 0.5 mM 8-Br-cAMP (Selleck Chemical) for 7 days, and the medium was replenished every 2 days. DMSO (equal volume to LDN57444), LDN57444 (dissolved in DMSO at 10mM) (indicated concentrations) or C188-2 (indicated concentrations) were added during the induction of decidualization in certain experiments.

### ELISA

In the in vitro decidualization assay, HESCs were treated with either DMSO or LDN57444. Then, the culture supernatant was collected at the indicated time points and centrifuged to eliminate cellular debris. The concentrations of PRL, CXCL12, IL15 and TGF-β were then analyzed according to standard protocols.

### Plasmid construct and cell transfection

To construct the UCHL1 knockdown shRNA plasmid, we cloned shRNA-encoding oligonucleotides into the lentiviral pLKO.1 puro vector, a gift from Bob Weinberg (Addgene plasmid # 8453; http://n2t.net/addgene:8453; RRID: Addgene_8453) [29], to target UCHL1 mRNA. The shRNA sequences were as follows: shUCHL1-1, 5ʹ-CGGGTAGATGACAAGGTGAAT-3ʹ, shUCHL1-2, 5ʹ-GTGTGAGCTTCAGA TGGTGAA-3ʹ, shUCHL1-3′ 5ʹ-CCAGCATGAGAACTTCAGGAA-3ʹ and scramble control 5ʹ-CCTAAGGTTAAGTCGCCCTCG-3ʹ, synthesized by Sangon Biotech.

For generation of the overexpression plasmid pLVX/UCHL1, the cDNA encoding the human UCHL1 gene was amplified from the cDNA of HESCs and inserted between the XhoI and XhaI sites of pLVX-IRES-ZsGreen1 (Clontech) and pCMV-Tag2B (Stratagene). The C90S mutation of UCHL1 used PCR-based site-directed mutagenesis based on pLVX/UCHL1 with the following primers: forward, 5ʹ-AATTCCTCTGGCACAATCGGACTTATTC-3ʹ, reverse 5ʹ-CGATTGTGCCAG AGGAATTCCCAATGG-3ʹ.

To establish UCHL1 knockdown or UCHL1-overexpressing HESCs, we performed lentivirus packaging and production according to the manufacturer’s protocol (http://www.addgene.org/tools/protocols/plko/). Briefly, lentiviral plasmids harboring the desired sequences were transfected into 293T cells, together with the packing plasmids pSPAX2 and pMD2G using Lipofectamine 2000 according to the reagent protocol. The supernatants were collected 48 h after transfection, filtered through a 0.45 µm filter and used to infect HESCs in a 6-well plate supplemented with 10 µg/mL polybrene (Thermo Fisher).

### Statistics

All data were shown as the mean ± SEM and were obtained from at least three independent experiments. Significant differences were analyzed by the Mann–Whitney U test, one-way ANOVA and two-way ANOVA with GraphPad Prism (version 8.0, GraphPad Software) and Statistical Package for Social Science software (version 23.0, SPSS).

## Results

### UCHL1 defects were related to impaired decidualization and decreased dNKs in patients with miscarriage

**Fig. 1:**
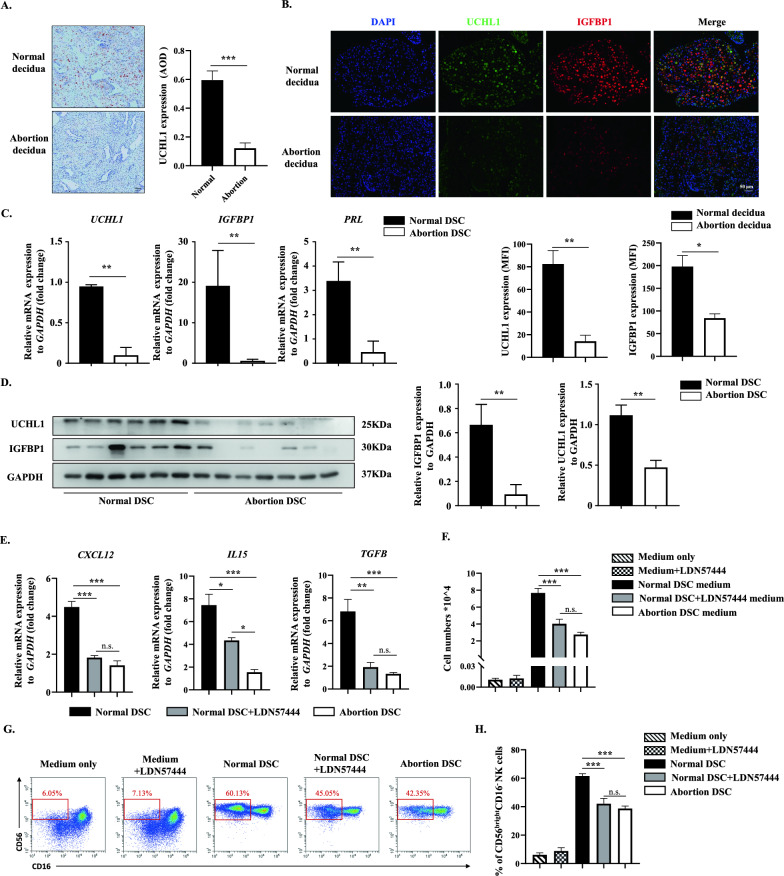
UCHL1 defects were related to impaired decidualization and decreased dNKs in patients with miscarriage. Decidual tissues from women with normal pregnancy (normal decidua) and miscarriage (abortion decidua) were collected to determine the expression profile of UCHL1 and decidual markers. **A** Immunohistochemical analysis was used to verify the expression of UCHL1 in normal decidua and abortion decidua and the average optical density (AOD) analysis of UCHL-1 was analyzed. **B** The expression of UCHL1 and IGFBP1 was analyzed by immunofluorescence in normal decidua and abortion decidua. The mean fluorescent intensity (MFI) of UCHL1 and IGFBP1 was quantified. DSCs were then isolated from decidual tissues of women with normal pregnancy (normal DSC) and miscarriage (abortion DSC), and the expression of decidual marker genes in DSCs was detected. **C** The expression levels of UCHL1 and the decidual markers IGFBP1 and PRL were determined by qPCR in normal DSCs (n = 20) and abortion DSCs (n = 20). **D** The protein levels of UCHL1 and IGFBP1 were determined by western blotting in normal DSCs (n = 6) and abortion DSCs (n = 7). **E** Twenty-four hours before mRNA was collected, normal DSCs were treated with LDN57444 (5 μM). The expression of CXCL12, IL-15 and TGF-β in normal DSCs, LDN57444-treated normal DSCs and abortion DSCs was then measured by qPCR. **F** The culture medium (CM) of normal DSCs, LDN57444-treated normal DSCs and abortion DSCs was collected. Then, these culture media were placed into the basolateral chambers of transwell inserts, with unconditioned medium (LDN57444 added or not) as the negative control, and the pNKs were placed in the apical chambers of the transwell system. Forty-eight hours after coculture, the transwell inserts were removed, pNKs that migrated into the basolateral chamber were counted, and the statistical analysis is shown. **G**, **H** pNKs were cocultured with normal DSCs, LDN57444-treated normal DSCs and abortion DSCs, with unconditioned medium (LDN57444 added or not) as the negative control, and 48 h later, the suspended cells in the coculture system were collected for flow cytometric analysis. The proportion of CD56^bright^CD16^−^ dNKs is shown in the red box (**G**), and the statistical analysis is shown in **H**. The results of the coculture assay are representative of four independent experiments and are represented by the mean ± SEM. Significant differences were determined by Mann‒Whitney U test (**C**, **D**) or one-way ANOVA (**E**, **F**, **H**) and are expressed as *P < 0.05, **P < 0.01, ***P < 0.001 and n.s. no significance

Aberrant endometrial decidualization is a major maternal cause of miscarriage [1] and frequently occurs in patients suffering pregnancy loss. Consistent with previous studies [27, 30, 31], we found that decidualization was impaired in abortion patients, as shown by abnormal morphology (Fig. 1A) and downregulation of the decidual markers IGFBP1 and PRL (Fig. 1B–D). By extension, the expression of UCHL1 was also decreased in the decidua from abortion patients (abortion decidua), as indicated by IHC staining (Fig. 1A) and immunofluorescence of UCHL1 (Fig. 1B). We then further isolated DSCs from the collected decidual tissues and examined UCHL1 expression levels. Similarly, the abortion DSCs also displayed decreased expression of UCHL1 at both the mRNA and protein levels (Fig. 1C, D).

Additionally, we found that CXCL12, but not CXCL9, CXCL10, or CXCL11 (Supplementary Fig. 1), which are critical chemokines expressed by DSCs for the recruitment of pNKs into the fetal-maternal interface [6], was significantly downregulated in abortion DSCs compared to normal DSCs (Fig. 1E). As a result, the ability of abortion DSCs to recruit pNKs was reduced (Fig. 1F). We then measured the expression of IL15 and TGF-β, which are key cytokines for the dNKs education [32], in DSCs from different samples and found that they were also downregulated in abortion DSCs (Fig. 1E). Consequently, the percentage of CD56^bright^CD16^−^ dNKs cocultured with abortion DSCs was significantly lower than that of dNKs cocultured with normal DSCs (Fig. 1G, H). Nevertheless, when pretreated with LDN57444, an inhibitor of UCHL1 [17], the expression of CXCL12, IL15 and TGF-β in normal DSCs was significantly decreased, as well as their ability to recruit and educate dNKs, which were similar to that of the abortion DSCs (Fig. 1E–H).

Collectively, these results indicated that UCHL1 was downregulated in the decidua from abortion patients, and the defect of UCHL1 was associated with the impaired decidualization and aberrant modulation of dNKs.

### Functional restriction of UCHL1 disrupted HESC decidualization and consequently reduced cytokines

**Fig. 2:**
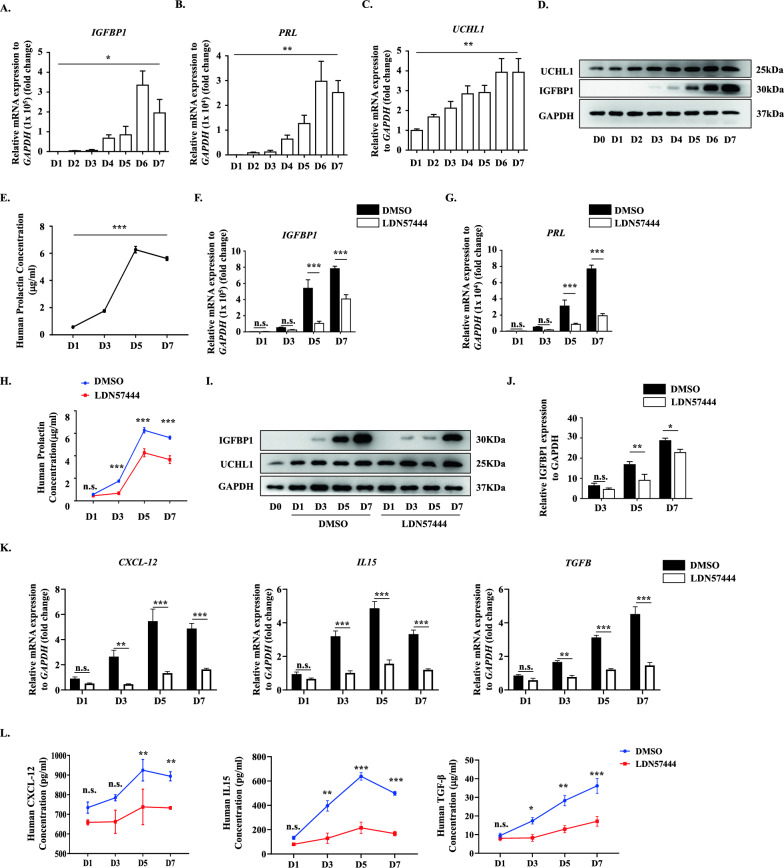
Functional restriction of UCHL1 disrupted HESC decidualization and consequently reduced cytokines. The effect of the UCHL1 inhibitor was determined in an in vitro model of decidualization. **A**–**C** qPCR analysis showed IGFBP1, PRL and UCHL1 expression during the decidualization of HESCs. **D** The protein levels of UCHL1 and IGFBP1 were analyzed by western blotting. **E** The concentration of PRL in the culture medium of HESCs undergoing decidualization was analyzed by ELISAs. **F**, **G** HESCs were treated with DMSO or LDN57444 (5 μM) on the day that decidualization was induced. The expression of the decidual markers IGFBP1 and PRL was detected by qPCR at D1, D3, D5 and D7 of decidualization. **H** The concentration of PRL was determined in DMSO- or LDN57444-treated HESC culture medium by ELISAs at D1, D3, D5 and D7 of decidualization. **I**, **J** The protein levels of IGFBP1 and UCHL1 in DMSO- or LDN57444-treated HESCs were detected via western blotting at D1, D3, D5 and D7 of decidualization, with D0 as the blank control, and the statistical analysis of relative IGFBP1 expression is shown in **J**. **K** The expression of CXCL12, IL15 and TGF-β by DMSO- or LDN57444-treated HESCs at D1, D3, D5 and D7 of decidualization was detected by qPCR. **L** The culture media of DMSO- or LDN57444-treated HESCs at D1, D3, D5 and D7 of decidualization were collected, and the concentrations of CXCL12, IL15 and TGF-β in the media were determined by ELISAs. The results are representative of three to four independent experiments and are represented by the mean ± SEM. Significant differences were analyzed by one-way ANOVA (**A**–**C**, **F**) or two-way ANOVA (**F**–**H**, **J**–**L**) and are expressed as *P < 0.05, **P < 0.01, ***P < 0.001 and n.s. no significance

To further affirm the function of UCHL1 in human decidualization, given the difficulty of collecting human decidual tissues at different stages, we employed an immortalized human endometrial stromal cell line (T-HESC) to establish the well-known in vitro model of decidualization [33]. In this model, HESCs underwent extensive stromal to decidual transformation in response to a cocktail of cAMP plus MPA for seven days, which mimics the decidual process in vivo. The model was constructed successfully, as indicated by increased expression of the decidual markers IGFBP1 and PRL at both the mRNA level (Fig. 2A, B) and protein level (Fig. 2D, E). Excitingly, UCHL1 showed progressively increased during HESC decidualization (Fig. 2C, D), indicating a positive regulatory role of UCHL1 in decidualization.

We then treated HESCs with the UCHL1 inhibitor LDN57444 during simulated in vitro decidualization to further verify its regulatory function in decidualization. The results showed that LDN57444 significantly reduced the mRNA levels of IGFBP1 and PRL in a dose-dependent manner (Supplementary Fig. 2A, B). Since 5 µM LDN57444 effectively inhibited decidualization of HESCs without causing substantial cell death, this concentration was used to subsequent experiments. We observed a significant decrease in the mRNA expression levels of the decidual markers IGFBP1 and PRL in the LDN57444-treated HESCs during simulated in vitro decidualization (Fig. 2F, G), as well as the other two decidual markers BMP2 and WNT 4[4] (Supplementary Fig. 2C). Moreover, western blots (Fig. 2I, J) demonstrated a decrease in IGFBP1 protein level upon treatment with LDN57444, while ELISAs displayed the downregulated expression of PRL in LDN57444-treated HESCs (Fig. 2H).

According to the results from patient samples, UCHL1 deficiency disrupted the decidual process, which further led to the decreased expression of CXCL12, IL15 and TGF-β (Fig. 1). Then, we wondered whether UCHL1 suppression also impaired chemokines and cytokines secretion in decidualized HESCs. As the data shown, mRNA levels of CXCL12, IL15 and TGF-β increased during the simulated in vitro decidualization. However, blockade of UCHL1 with LDN57444 also prevented the increase in these cell factors (Fig. 2K). Consistently, reduced concentrations of CXCL12, IL15 and TGF-β were detected in the culture medium of LDN57444-treated decidualized HESCs (Fig. 2L).

These data confirmed the role of UCHL1 in maintaining successful decidualization and expression of cell factors by decidualized HESCs.

### Genetic deletion of UCHL1 induced decidualization failure along with aberrant education of dNKs

**Fig. 3:**
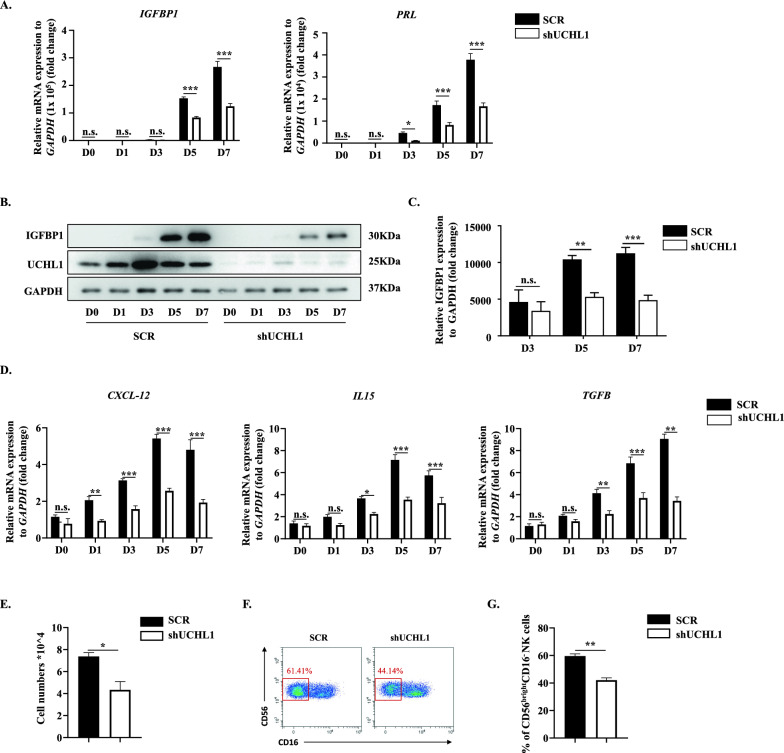
Genetic deletion of UCHL1 induced decidualization failure along with aberrant education of dNKs. UCHL1 was knocked down in HESCs to verify its function in decidualization and dNK modulation. **A** HESCs were transfected with lentivirus expressing shRNA targeting UCHL1 (shUCHL1) or scramble shRNA (SCR). The expression of IGFBP1 and PRL in SCR or shUCHL1 HESCs at D0, D1, D3, D5 and D7 of decidualization was detected by qPCR. **B**, **C** The protein levels of IGFBP1 and UCHL1 in SCR or shUCHL1 HESCs at D0, D1, D3, D5 and D7 of decidualization were determined by western blotting (**B**), and the statistical analysis of relative IGFBP1 expression is shown in **C**. **D** The expression of CXCL12, IL15, and TGF-β in SCR or shUCHL1 HESCs at D0, D1, D3, D5 and D7 of decidualization was detected by qPCR. **E** In the transwell assay, the pNKs that migrated into the basolateral chamber containing culture medium (CM) of SCR or shUCHL1 HESCs collected at D7 of decidualization were counted, and the statistical analysis is shown. **F**, **G** pNKs were cocultured with SCR or shUCHL1 HESCs at D7 of decidualization, and 48 h later, the suspended cells in the coculture system were collected for flow cytometric analysis. The proportion of CD56^bright^CD16^−^ dNKs is shown in the red box (**F**), and the statistical analysis is shown in **G**. The results are representative of three to five independent experiments and are represented by the mean ± SEM. Significant differences were analyzed by two-way ANOVA (**A**, **C**, **D**) or Mann‒Whitney U test (**E**, **G**) and are expressed as *P < 0.05, **P < 0.01, ***P < 0.001 and n.s. no significance

Despite being more sensitive to UCHL1 inhibition, LDN57444 also suppresses the activity of UCHL3 [34]. To exclude the possible interference from UCHL3, we designed three specific shRNAs for knocking down UCHL1 in HESCs. We found that shUCHL1-3 was the most efficient in depleting UCHL1 (Supplementary Fig. 3A, B); therefore, we selected it for subsequent experiments (referred to as shUCHL1). Consistent with the effects of the UCHL1 inhibitor, the decidual marker IGFBP1 was remarkably downregulated in shUCHL1-HESCs during the process of in vitro-induced decidualization at both the mRNA and protein levels (Fig. 3A–C) compared to the scramble-transfected cells (SCR), as well as PRL (Fig. 3A). In addition, the expression of CXCL12, IL15 and TGF-β was significantly decreased in decidualized shUCHL1-HESCs (Fig. 3D), resulting in inhibited recruitment and education of dNKs, as indicated by reduced cell numbers migrating into the basolateral chamber in the transwell assay (Fig. 3E) and a decreased percentage of CD56^bright^CD16^−^ dNKs (Fig. 3F, G).

These data further proved that UCHL1, but not UCHL3, regulated the decidual process and that UCHL1 deficiency led to impaired decidualization as well as reduced dNKs.

### UCHL1 positively regulates HESCs decidualization dependent on its deubiquitinating activity

**Fig. 4:**
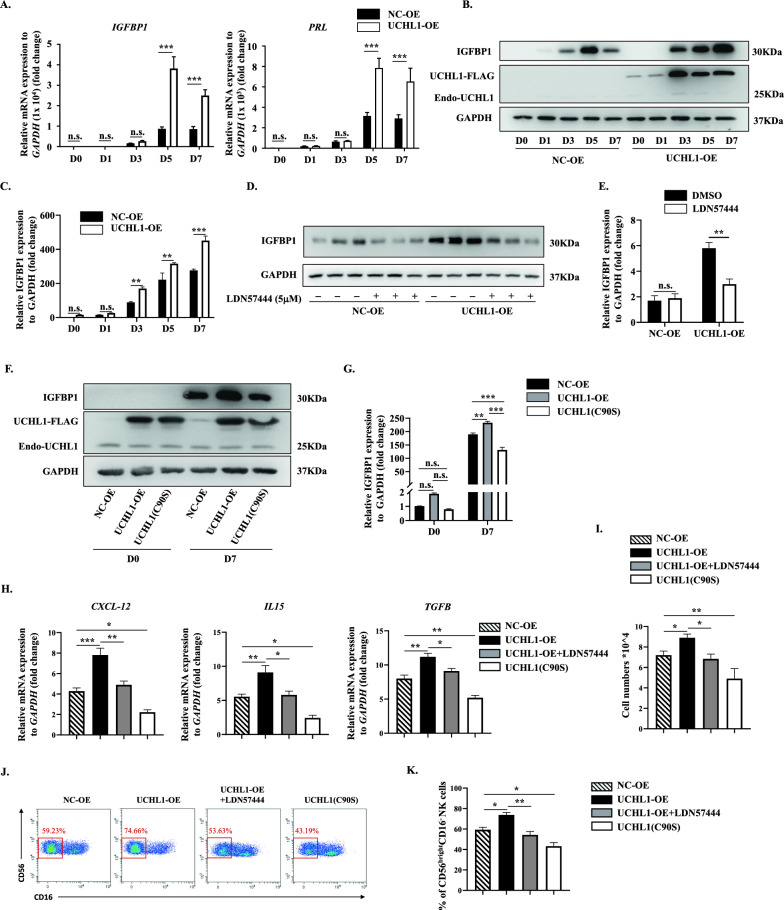
UCHL1 positively regulates HESCs decidualization dependent on its deubiquitinating activity. UCHL1 was overexpressed in HESCs to detect the effect on decidualization and dNK modulation. **A**–**C** HESCs were infected with pLVX-IRES-zsGreen1-UCHL1 (UCHL1-OE) or pLVX-IRES-zsGreen1 (NC-OE) lentivirus, and GFP^+^ HESCs were sorted by FACS. Then, proteins and mRNAs of UCHL1-OE or SCR HESCs at D0, D1, D3, D5 and D7 of decidualization were collected. The expression of IGFBP1 and PRL at the mRNA level was detected by qPCR (**A**). The protein levels of IGFBP1, overexpressed UCHL-FLAG and Endo-UCHL1 were detected by western blotting (**B**), and the statistical analysis is shown in **C**. **D**, **E** NC-OE or UCHL1-OE HESCs were treated with DMSO or LDN57444 (5 μM), and proteins were collected at D7 of decidualization. The expression of IGFBP1 was detected by western blotting (**D**), and the statistical analysis is shown (**E**). **F**, **G** HESCs were transfected with lentivirus containing vectors that overexpressed the mutated UCHL1 (C90S), and proteins from NC-OE, UCHL1-OE, UCHL1 (90S) were collected at D0 and D7 of decidualization. The expression of IGFBP1, overexpressed UCHL-FLAG and Endo-UCHL1 was detected by western blot (**F**), and the statistical analysis is shown in **G**. **H**–**J** The expression of CXCL12, IL15, and TGF-β in NC-OE, UCHL1-OE, and UCHL1-OE HESCs treated with LDN57444 (UCHL1-OE + LDN57444) or UCHL1 (C90S) at D7 of decidualization was detected by qPCR. **K** In the transwell assay, the pNKs that migrated into the basolateral chamber containing culture medium (CM) of NC-OE, UCHL-OE, UCHL1-OE treated with LDN57444 (UCHL1-OE + LDN57444) and UCHL1 (C90S) HESCs collected at D7 of decidualization were counted, and the statistical analysis is shown. **I**, **M** pNKs were cocultured with NC-OE, UCHL-OE, UCHL1-OE cells treated with LDN57444 (UCHL1-OE + LDN57444) and UCHL1 (C90S) HESCs at D7 of decidualization, and 48 h later, the suspended cells in the coculture system were collected for flow cytometric analysis. The proportion of CD56^bright^CD16^−^ dNKs is shown in the red box (**I**), and the statistical analysis is shown in **M**. The results are representative of three to five independent experiments and are represented by the mean ± SEM. Significant differences were analyzed by two-way ANOVA (**A**, **C**, **E**, **G**) or one-way ANOVA (**H**–**K**, **M**) and are expressed as *P < 0.05, **P < 0.01, ***P < 0.001 and n.s. no significance

Since UCHL1 defects have a negative impact on decidualization, we wondered if overexpressing UCHL1 would be beneficial for the decidual process. As expected, UCHL1 overexpression (Supplementary Fig. 4A, B) induced a dramatic increase in IGFBP1 and PRL in HESCs during simulated decidualization in vitro (Fig. 4A–C). Likewise, the expression of CXCL12, IL15 and TGF-β by decidualized HESCs was also upregulated when UCHL1 was overexpressed (Fig. 4H). In the transwell assay, more pNKs were recruited into the basolateral chamber containing culture medium from UCHL1-overexpressing (UCHL1-OE) HESCs collected on Day 7 of simulated decidualization in vitro (Fig. 4I). Furthermore, the proportion of CD56^bright^CD16^−^ dNKs increased to approximately 75% after coculture with induced-decidualized UCHL-OE HESCs, whereas there was only 60% of dNKs in the coculture assay with decidualized NC-OE HESCs (Fig. 4J, K). Nevertheless, the enhancing effects of UCHL1 overexpression on HESC decidualization and dNK modulation were reversed by treatment with LDN57444 (Fig. 4D–E, H–K), which confirmed the positive role of UCHL1 in regulating HESC decidualization.

UCHL1 is a deubiquitinating enzyme that efficiently hydrolyzes small C-terminal adducts of ubiquitin to remove ubiquitin from proteins, thereby inhibiting protein degradation [35]. In addition, UCHL1 has a high affinity for the monomeric ubiquitin-like molecule NEDD8 but does not hydrolyze it, indicating that the function of UCHL1 is not solely dependent on its deubiquitinating activity [36]. To confirm whether UCHL1-mediated regulation of decidualization depended on enzymatic activity, we overexpressed a mutated UCHL1 at a cysteine residue (C90S) that lost its deubiquitinating activity in HESCs. We found that decidualization was inhibited in these cells, as evidenced by decreased IGFBP1 expression compared to NC-OE-HESCs (Fig. 4F, G). Moreover, the expression of CXCL12, IL15 and TGF-β was significantly reduced with UCHL1 (C90S) overexpression (Fig. 4H), which consequently impaired the recruitment and education of dNKs by decidualized HESCs (Fig. 4I–K). These data suggested that the promotion of decidualization by UCHL1 was dependent on its deubiquitinating activity.

In conclusion, UCHL1 positively regulates HESC decidualization in a manner that relied on its deubiquitinating enzymatic activity.

### UCHL1 promoted decidualization mediated by activation of JAK2/STAT3 signaling

**Fig. 5:**
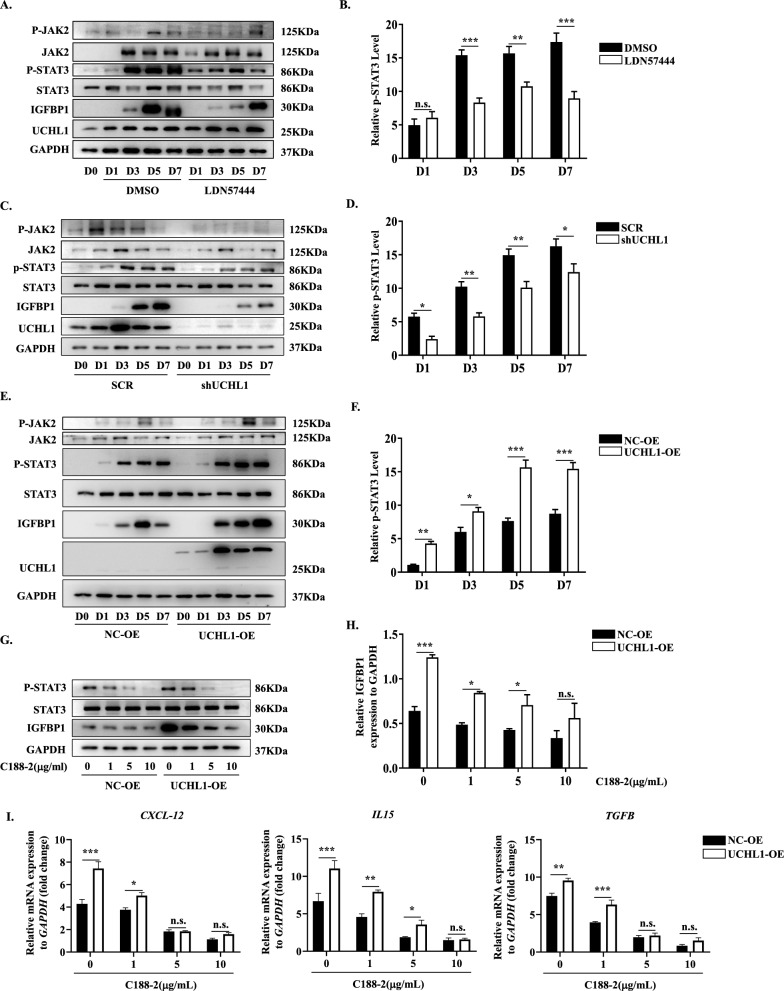
UCHL1 promoted decidualization mediated by activation of JAK2/STAT3 signaling. The activation of JAK2-STAT3 signaling in HESCs was analyzed during decidualization. **A** The main signaling pathways were determined in DMSO- or LDN57444-treated HESCs at different days of decidualization by western blotting, and the relative phosphorylation level of STAT3 was normalized to total STAT3 and GAPDH (**B**). **C** The JAK2-STAT3 signaling components JAK2 and STAT3 were determined in SCR or shUCHL1 HESCs at different days of decidualization, and the relative phosphorylation level of STAT3 was normalized to total STAT3 and GAPDH (**D**). **E** The JAK2-STAT3 signaling components JAK2 and STAT3 were analyzed in NC-OE or UCHL1-OE HESCs at different days of decidualization, and the relative phosphorylation level of STAT3 was normalized to total STAT3 and GAPDH (**F**). The decidual marker IGFBP1 (**G**, **H**) and STAT3 signaling (**G**) were analyzed in NC-OE or UCHL1-OE HESCs at D7 of decidualization in the presence of the STAT3 inhibitor C188-2. **I** mRNAs were collected from NC-OE or UCHL1-OE HESCs treated with the indicated concentration of C188-2 at D7 of decidualization, and the expression of CXCL12, IL15 and TGF-β was detected by qPCR. The results are representative of three to four independent experiments and are represented by the mean ± SEM. Significant differences were analyzed by two-way ANOVA (**B**, **D**, **F**, **H**, **I**) and are expressed as *P < 0.05, **P < 0.01, ***P < 0.001 and n.s. no significance

To elucidate the molecular mechanisms underlying UCHL1 regulation of decidualization, we examined the main transcription factors and signaling pathways involved in decidualization. As shown, the transcription factor FOXO1 and its upstream signaling pathway ERK, which play critical roles in regulating cell differentiation during decidualization [27], were not affected by LDN57444 treatment (Supplementary Fig. 5). However, as an essential signaling pathway in regulating decidualization [26], the phosphorylation levels of JAK2 and STAT3 were decreased in the LDN57444-treated HESCs during decidualization, a similar trend as IGFBP1 (Fig. 5A, B). UCHL1 knockdown exhibited a conformable effect on the activation of JAK2/STAT3 signaling in response to LDN57444 treatment (Fig. 5C, D). In contrast, overexpression of UCHL1 activated JAK2-STAT3 signaling, as evidenced by the enhanced phosphorylation levels of JAK2 and STAT3 during decidualization (Fig. 5E, F). These data indicated that the promotion of decidualization by UCHL1 might be attributed to the activation of the JAK2/STAT3 signaling pathway. To confirm this possibility, we applied C188-2 to specifically inhibit the activity of STAT3 in UCHL1-OE HESCs and found that the positive effect of UCHL1 overexpression on IGFBP1 expressio was dose-dependently inhibited by C188-2 (Fig. 5G, H), as well as the expression of CXCL12, IL15 and TGF-β (Fig. 5I). Hence, these data suggested that the promotion of UCHL1 on HESC decidualization and cytokine production was mediated by activating the JAK2-STAT3 pathway.

### Blockage of UCHL1 led to aberrant decidualization in mice

**Fig. 6:**
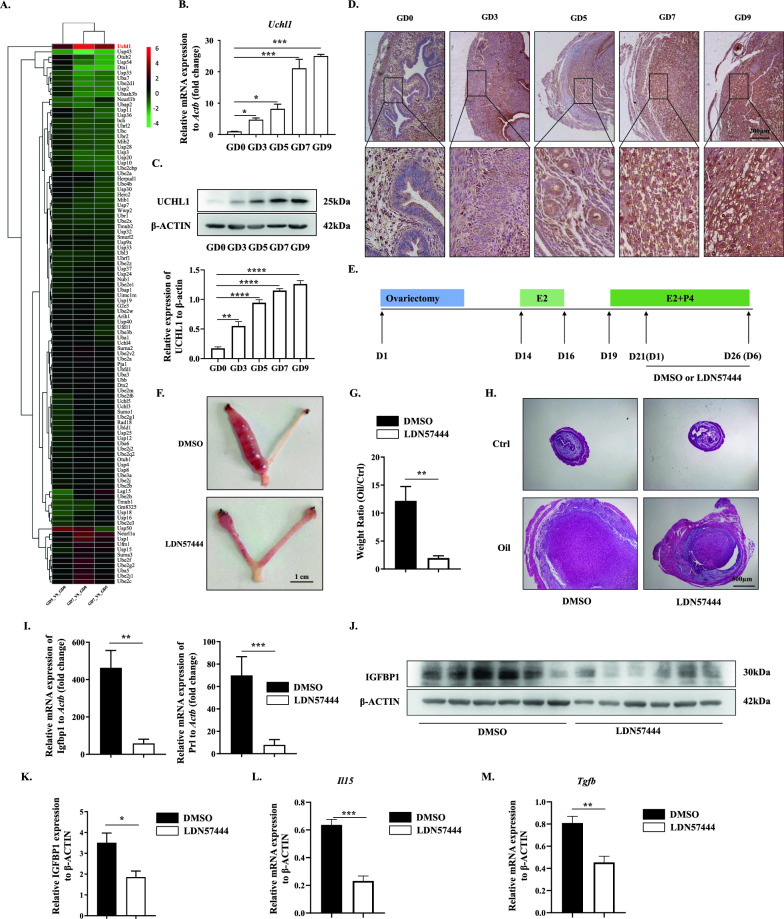
Blockage of UCHL1 led to aberrant decidualization in mice. **A**–**D** The expression of the deubiquitinating enzyme UCHL1 was determined in endometrial decidualization during early pregnancy in mice. **A** Heatmap showing the change in ubiquitin-related gene sets in the endometrium of normal pregnant mice at GD0, GD5 and GD7 by RNA-seq. **B**, **C** qPCR and Western blot analysis of UCHL1 expression in the endometrium of normal pregnant mice at GD0, GD3, GD5, GD7 and GD9 (n = 6). The statistical analysis of the relative expression of UCHL1 to β-actin was shown. **D** Immunohistochemical analysis of UCHL1 in the endometrium of normal pregnant mice at GD0, GD3, GD5, GD7 and GD9. The expression of UCHL1 was analyzed in HESC in vitro decidualization induced by 0.5 mM cAMP and 10 µM MPA. **E**–**M** An artificial decidualization mouse model was established to determine the effect of the UCHL1-specific inhibitor LDN57444 on in vivo decidualization. **E** Scheme of the sesame oil-induced artificial decidualization procedure. **F** Representative photographs show the morphology of sesame oil-induced uterine horns from the control or LDN57444-treated mice collected 6 days after sesame oil induction. **G** The ratio between the weight of the sesame oil-induced horn and the weight of the contralateral uninduced horn was measured for each control or LDN57444-treated mouse. **H** H&E staining showed the histology of unstimulated control and stimulated horns in the DMSO- or LDN57444-treated mice at Day 6. **I** The expression of decidual markers IGFPB1 and PRL was measured by qPCR in the endometrium of the DMSO- or LDN57444-treated mice. **J**, **K** Western blotting was used to detect the protein level of IGFBP1 in the endometrium of the DMSO- or LDN57444-treated mice (**J**), and the statistical analysis is shown in **K**. **L**, **M** The expression of IL15 and TGF-β was detected by qPCR in the endometrium of the DMSO- or LDN57444-treated mice. The results are representative of four to six independent experiments and are represented by the mean ± SEM. Significant differences were analyzed by one-way ANOVA (**B**) or the Mann‒Whitney U test (**G**, **I**, **K**‒**M**) and are expressed as *P < 0.05, **P < 0.01, ***P < 0.001, ****P < 0.0001

In contrast to human decidualization, which occurs throughout the endometrium even in the absence of a conceptus, decidual changes in mice are dependent on the presence of an embryo in the uterus. Although human and murine decidua have similar functions, their gene expression profiles are quite different from each other [37]. These differences prompted us to investigate whether UCHL1 played a consistent role in the murine decidual process as it does in human decidualization. To explore this possibility, we performed RNA sequencing (RNA-seq) of mouse endometrial samples with normal pregnancy at three different gestational days (GD): GD0 when endometrial decidualization had not occurred, GD5 when decidualization was initiated by embryo attachment, and GD7 when decidual tissues spread surrounding the embryo [37]. Among the differentially expressed ubiquitination-related genes among GD0, GD5 and GD7, UCHL1 was the gene that changed most. Along with the decidual process, UCHL1 expression progressively increased (Fig. 6A). Consistent with the RNA-seq analysis, the expression of UCHL1 was continuously upregulated during decidualization at both the mRNA level (Fig. 6B) and the protein level (Fig. 6C), which was similar to the results obtained from the in vitro decidualization model (Fig. 2C, D). IHC staining of these decidual tissues from different time points of GD further validated the upregulation of UCHL1 in the process of murine decidualization (Fig. 6D).

We then explored whether UCHL1 also positively regulated the decidual process in mice. To this end, we employed an artificially induced decidualization mouse model (Fig. 6E). As previously reported [38], after ovariectomy, mice were treated with estrogen (E2) and progesterone (P4), and one side of the uterine cavity was stimulated by sesame oil to induce artificial decidualization that mimicked embryo implantation, while the contralateral side served as a control. On the day when artificial decidualization was induced, LDN57444 or DMSO was injected i.p. to the mice. As shown in Fig. 6F, sesame oil-induced deciduomas in the uterus of the LDN57444-treated mice were much smaller than those in the DMSO-treated mice. Measurement of the weight of the uterine horn (Fig. 6G) and HE staining of the deciduoma (Fig. 6H) confirmed the negative effect of LDN57444 on decidualization. Additionally, both mRNA level (Fig. 6I) and protein level (Fig. 6J, K) showed downregulation of the decidual marker IGFBP1 in the deciduoma of LDN57444-treated mice compared to control, as well as another decidual marker PRL (Fig. 6I). Furthermore, there was a significant decrease in expression of IL15 and TGF-β, cytokines involved in educating murine dNKs in the decidua [39, 40], in the deciduoma of LDN57444-treated mice (Fig. 6L, M). Taken together, these results provided further evidence that UCHL1 also acted as a protector of decidualization in mice, and its blockade impaired decidualization along with reduced cytokine secretion.

### Inhibition of UCHL1 resulted in miscarriage in mice

**Fig. 7:**
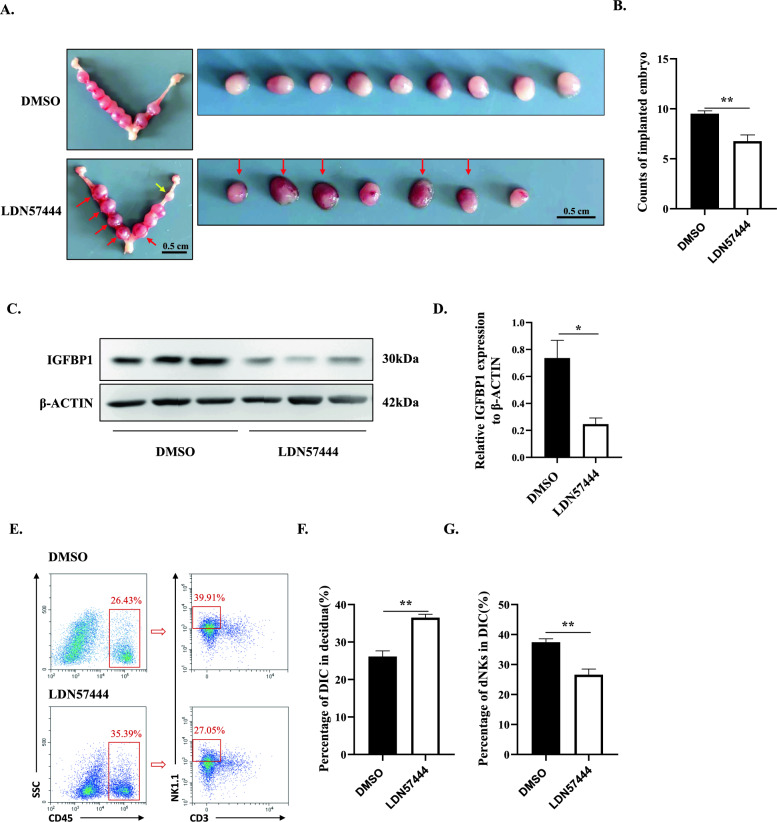
Inhibition of UCHL1 resulted in miscarriage in mice. **A** Representative images of the uterus and fetus in the uterus of GD8 female mice treated with DMSO or LDN57444. Red arrows: placenta with hemorrhage. Yellow arrow: resorption implantation site. **B** The mice were treated with DMSO or LDN57444 during pregnancy, and the number of embryos implanted at GD8 was quantified. **C** Uterine tissues from pregnant mice treated with DMSO or LDN57444 were collected, and western blotting was performed to detect the expression of IGFBP1. The statistical analysis is shown in **D**. **E** Flow cytometric analysis was used to detect the proportion of dNKs in the uterus of pregnant mice at GD8 treated with DMSO or LDN57444. The CD45^+^ DICs and CD45^+^CD3^−^NK1.1^+^ dNKs are emphasized in the red box. The statistical analysis of the DIC percentage in decidua and the dNK percentage in DICs is displayed in **F** and **G**. The results are representative of three to four independent experiments and are represented by the mean ± SEM. Significant differences were determined by the Mann‒Whitney U test (**B**, **D**, **F**, **G**) and are expressed as *P < 0.05, **P < 0.01

To further investigate the roles of UCHL1 in miscarriage related to aberrant decidualization, we treated pregnant mice with corn oil containing DMSO or LDN57444 from GD5 to 7 when murine ESCs undergo decidualization [37], and on GD8, the pregnancy outcome was observed. As shown in Fig. 7A, LDN57444-treated mice exhibited a severe miscarriage sign, while the DMSO-treated mice presented a healthy pregnancy. Moreover, LDN57444 treatment significantly reduced the number of implanted embryos (Fig. 7B). Then, we examined whether the UCHL1 inhibition-induced abortion symptoms were due to decidualization failure. To this end, we collected decidual tissues surrounding the site of embryo implantation and examined the expression of the decidual marker IGFBP1 and the percentage of CD45^+^CD3^−^NK1.1^+^ murine dNKs [41]. As shown in Fig. 7C and D, the expression of IGFBP1 was significantly decreased in decidua from the LDN57444-treated mice, suggesting that UCHL1 blockade induced impaired decidualization. In addition, intervention UCHL1 with LDN57444 led to a decrease in the percentage of dNKs in DICs (Fig. 7E, G), although the percentage of CD45^+^ DICs was increased in decidua from the LDN57444-treated mice (Fig. 7E, F), indicating that UCHL1 deficiency not only disrupted dNK modulation but also enhanced inflammation at the fetal-maternal interface, which collaboratively contributed to miscarriage. Overall, these findings demonstrated that UCHL1 inhibition resulted in miscarriage, which was attributed to decidualization failure along with defects in dNK modulation as well as enhancement of inflammation.

## Discussion

Miscarriage is a prevalent complication in human pregnancy, and apart from affecting the course of pregnancy, it also exhibits enduring implications on both physical and psychological well-being. It has been shown that miscarriage leads to an increased risk of cardiovascular diseases and venous thromboembolism [1]. In addition, emotional disorders, such as anxiety, depression and suicide, exhibit a robust correlation with miscarriage [42]. Given the multifaceted implications arising from miscarriage, this phenomenon necessitates thorough investigation and resolution as a significant societal and scientific concern. In the present study, we found that UCHL1, a deubiquitinating enzyme that regulates ubiquitinating and deubiquitinating dynamics, was significantly decreased in decidual tissue obtained from patients with miscarriage, highlighting the critical role of UCHL1 in maintaining normal pregnancy. Pharmacological inhibition of UCHL1 in pregnant mice resulted in severe symptoms of miscarriage, firmly confirming its protective role in pregnancy. Additionally, previous studies have demonstrated that *Uchl1*^−/−^ mice could only be obtained by intercrosses of *Uchl1*^±^ mice [43], suggesting that complete knockout of UCHL1 impair fertility. All these discoveries indicate that UCHL1 is a crucial regulator in pregnancy, and its defects contribute to miscarriage.

Defects arising in decidualization are notable contributors to adverse pregnancy outcomes, especially miscarriage [3, 44]. Therefore, we explored whether UCHL1 blockage-induced miscarriage was due to impaired decidualization. Previous studies have shown that UCHL1 is specifically expressed in decidual cells within uterus during early pregnancy [24], and consistently, our findings indicate that the expression of UCHL1 was progressively increased during decidualization in both mice and humans, indicating a positive regulatory role of UCHL1 in this process. In human samples, we observed downregulation of UCHL1 in DSCs isolated from the decidua of miscarriage patients, accompanied by impaired decidualization. Moreover, blockage of UCHL1 in pregnant mice led to insufficient decidualization. These findings suggested that defects in UCHL1 were associated with aberrant decidualization. We then further verified the role of UCHL1 in decidualization with an artificially induced decidualization mouse model and a simulated in vitro decidualization cell model and confirmed that UCHL1 positively regulates decidualization both in mice and in humans. The conserved role of UCHL1 in murine and human decidualization made it feasible to identify the pharmaceutical effect of drugs targeting UCHL1 on pregnancy-related diseases caused by abnormal decidualization using a mouse model, despite the difference in decidualization between mice and humans.

In addition to providing a nutritive “ground” for embryo implantation, decidualization also contributes to the establishment of immune tolerance at the maternal–fetal interface [45]. Generally, chemokines secreted by DSCs recruit immune cells such as pNKs, dendritic cells (DCs), macrophages and T cells into the decidua, and the cytokines expressed by DSCs further educate these immune cells into Th2-type DICs [6]. dNKs are the most abundant leukocyte population in the maternal–fetal interface, and these cells are mainly recruited from peripheral blood during the first trimester of human pregnancy [46]. Homing of pNKs into the uterus is dependent on CXCL12 secreted by DSCs, whereas the retention of pNKs in decidua occurs through DSC-derived CXCL9, CXCL10 and CXCL11 [7]. After recruitment, pNKs are educated in the environment established by DSCs. IL15 and TGF-β, which are both expressed in the decidua, promote the conversion of CD56^dim^CD16^+^ pNKs to CD56^bright^CD16^−^ dNKs [9, 47]. To evaluate whether UCHL1 regulates the recruitment and education of dNKs by DSCs, we initially examined the expression profile of chemokines and cytokines in DSCs derived from normal pregnancy or abortion patients. Notably, CXCL12, IL15 and TGF-β were significantly downregulated in abortion DSCs, consequently leading to a substantial decrease in dNK percentage in the coculture assay with abortion DSCs. Furthermore, inhibition of UCHL1 in normal DSCs recapitulated the phenotype of abortion DSCs, underscoring the pivotal role played by UCHL1 in maintaining dNK modulation by DSCs. UCHL1 also positively regulated CXCL12, IL15 and TGF-β expression in decidualized HESCs and the recruitment and education of dNKs by decidualized HESCs. Finally, using the pregnant mouse model and an artificially induced decidualization mouse model, we verified that blockage of UCHL1 using its inhibitor reduced the percentage of dNKs in murine DICs and impaired the expression of IL15 and TGF-β in the decidua. Collectively, we provide evidence that the progressively increased expression of UCHL1 during decidualization acts as a protector to maintain the recruitment and education of dNKs by DSCs. Nevertheless, considering the lack of precise targeting of pharmacological inhibition towards decidua or endometrial stroma, our further research will focus on developing a UCHL1-conditional knockout mouse model specific to the endometrial stroma in order to validate the specific impact of UCHL1 deficiency on decidualization and dNKs modulation.

Our findings further elucidate the molecular mechanism underlying UCHL1-mediated promotion of decidualization, as well as the modulation of dNKs by DSCs. The JAK2/STAT3 signaling pathway emerges as a pivotal regulator of decidualization in response to estrogen and progesterone [26]. In addition, STAT3 signaling regulates the expression of CXCL12 [48] and TGF-β [49], cell factors secreted by DSCs to recruit and educate dNKs. In our study, we found that JAK2/STAT3 signaling was activated during decidualization and inhibited when UCHL1 was blocked by an inhibitor or shRNA. Conversely, UCHL1 overexpression enhanced the activation of JAK2/STAT3 signaling during the decidual process. Moreover, after inhibition of STAT3 using its specific inhibitor, the increased expression of IGFBP1, CXCL12, IL15 and TGF-β resulting from UCHL1 overexpression was abolished. These results suggest that the promotion of UCHL1 on decidualization and modulation of dNKs by DSCs is dependent on the activation of JAK2/STAT3 signaling.

UCHL1 is a deubiquitinating enzyme, and as its name implies, the function of UCHL1 relies on its C-terminal hydrolase to remove ubiquitin from proteins and thus inhibit protein degradation via the proteasome [35]. To verify whether the role of UCHL1 in decidualization was dependent on its deubiquitinating enzymatic activity, we generated a mutated UCHL1 (C90S) lacked deubiquitinating functionality [50] and found that the decidualization induced in vitro was significantly inhibited, so was the production of CXCL12, IL15 and TGF-β. As a result, the percentage of dNKs was also decreased when cocultured with decidualized UCHL1(C90S)-HESCs. These data suggest that the deubiquitinating ability of UCHL1 is essential for its function in endometrial decidualization. However, it remains unclear which molecule is stabilized by UCHL1 during the process of promoting decidualization. As previously reported, during decidualization, E2 elicits uterine stromal IGF1 to activate STAT3 in the epithelium, thus promoting epithelial transformation into the decidual type [51]. This research suggests that UCHL1 may regulate decidualization by stabilizing IGF1 signaling and subsequently activating activates STAT3. Another hypothesis derives from the high affinity of UCHL1 for the monomeric ubiquitin-like molecule NEDD8 [36], a monomeric ubiquitin-like molecule, as neddylation is proposed to be essential for endometrial decidualization mediated by NEDD8 [33], indicating that UCHL1 may promote decidualization through regulating neddylation. Further investigations are warranted to elucidate and validate the precise molecules or signaling pathways involved in UCHL1-mediated regulation of decidualization.

## Conclusions

In conclusion, our findings demonstrated that UCHL1 deficiency was a critical cause of miscarriage, which was due to aberrant decidualization and impaired dNK modulation. The present study not only enriches the basic knowledge of the etiology of miscarriage but also provides a new intervention target to diagnose and treat miscarriage.

### Supplementary Information

Supplementary material 1.

Supplementary material 2.

## Data Availability

The RNA-seq datasets of mouse endometrium samples generated during the current study are available in the supplementary information.
