# Supplementary material for: Deficiency of UCHL1 results in insufficient decidualization accompanied by impaired dNK modulation and eventually miscarriage
Source: J Transl Med. 2024 May 20;22:478. doi: 10.1186/s12967-024-05253-0 (PMC11103838; doi:10.1186/s12967-024-05253-0)
Supplement: Supplementary file 1 — Supplementary material 1. [file 12967_2024_5253_MOESM1_ESM.pdf]

Supplementary figure legends

Supplementary Figure 1. The expression of chemokines CXCL9, CXCL10 and CXCL11 were not affected in abortion DSCs.

mRNA was collected from Normal DSC and Abortion DSC, and the expression of CXCL9, CXCL10 and CXCL11 was detected by Q-PCR. The results are representative of four independent experiments and are represented by mean SEM. Significant differences were analyzed by two-way ANOVA, and are expressed as n.s. no significance.

Supplementary Figure 2. UCHL1 inhibitor LDN57444 suppressed the decidualization in a dose-dependent manner.

(A) HESCs were treated with different doses of LDN57444 on the day when decidualization was induced. mRNA from HESCs at D3 of decidualization was collected and the expression level of decidual markers IGFBP1 and PRL was detected by Q-PCR. **(B)** Protein from indicated dose of LDN57444-treated HESCs at D3 of decidualization was collected and the protein level of decidual markers IGFBP1 was detected by western blot. **(C)** In the *in vitro* decidualization assay, HESCs were treated with DMSO or LDN57444 (5μM). mRNAs were collected from HESCs at D1, D3, D5, D7 of decidualization, and the expression of BMP2 and WNT4 was determined by Q-PCR. The results are representative of four independent experiments and are represented by mean ±SEM. Significant differences were analyzed by one-way ANOVA **(A)**, or two-way ANOVA **(C),** and are expressed as ** $P<0.01$, * * * $P<0.001$, * * * * $P<0.0001$.

Supplementary Figure 3. UCHL1 was knocked down efficiently with shRNAs in HESCs.

(A, B) HESCs were transfected with lentivirus expressing shRNA targeting UCHL1 (shUCHL1-1, shUCHL1-2, shUCHL1-3) or scramble shRNA (SCR). The expression level of UCHL1 was measured by Q-PCR **(A)** and western blot **(B)** in shUCHL1(shUCHL1-1, shUCHL1-2, shUCHL1-3) HESCs or SCR-HESCs. The results are representative of four independent experiments and are represented by mean ±SEM. Significant differences were analyzed by one-way ANOVA **(A)**, and are expressed as ** $P<0.01$.

Supplementary Figure 4. UCHL1 was successfully overexpressed in HESCs.

(A, B) HESCs were infected with pLVX-IRES-zsGreen1-UCHL1 (UCHL1-OE) or pLVX-IRES-zsGreen1 (NC-OE) lentivirus, and the GFP+ HESCs were sorted by FACS. The expression of UCHL1 was measured by Q-PCR **(A)** and western blot **(B)**. The results are representative of four independent experiments and are represented by mean ±SEM. Significant differences were analyzed by Mann-Whitney U test **(A)** and are expressed as *P<0.05.

Supplementary Figure 5. The activation of ERK/FOXO1 was uninfluenced by UCHL1 inhibitor during decidualization.

The activation of ERK/FOXO1 signaling pathways were determined in DMSO or LDN57444 treated HESCs at indicated days of decidualization by western blot.

Supplementary figure 1

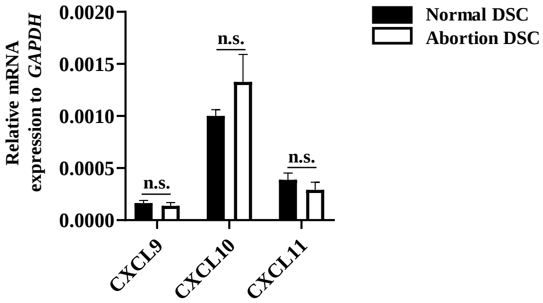

Supplementary figure 2

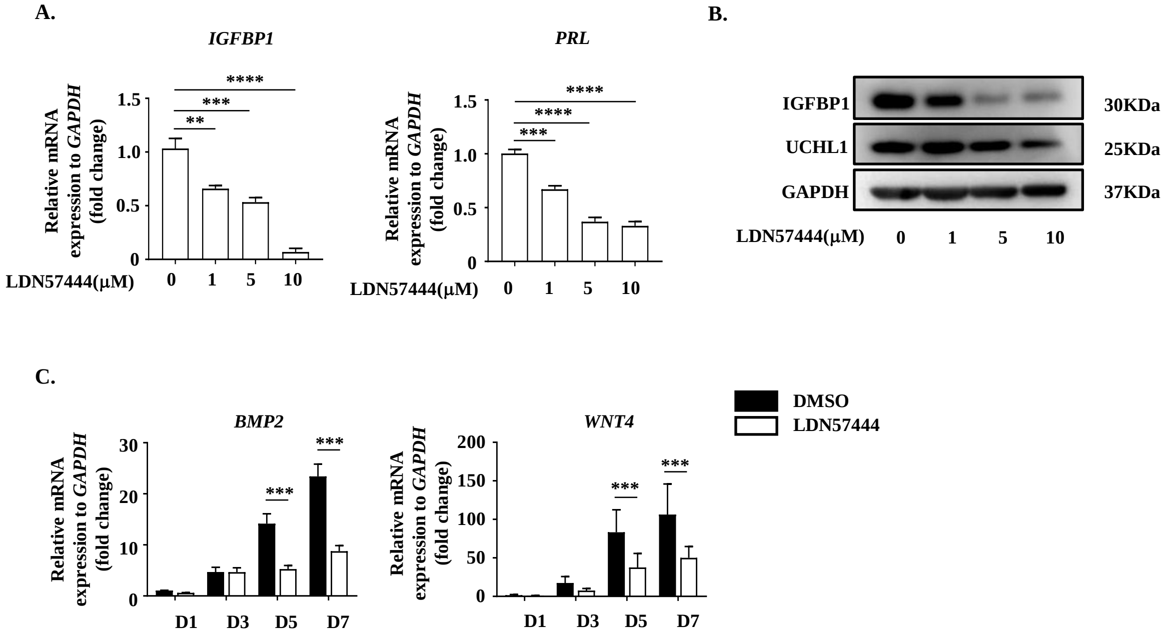

Supplementary figure 3

A.

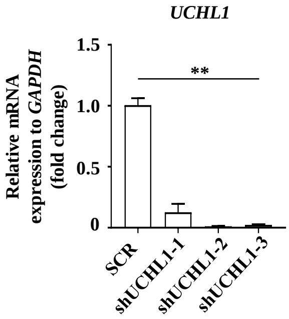

B.

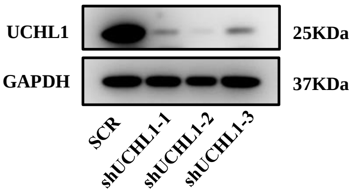

Supplementary figure 4

A.

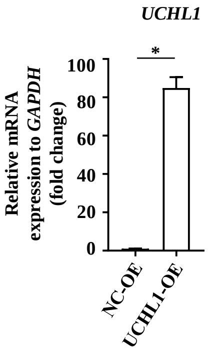

B.

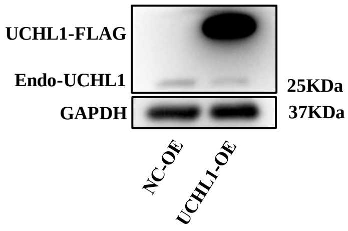

Supplementary figure 5

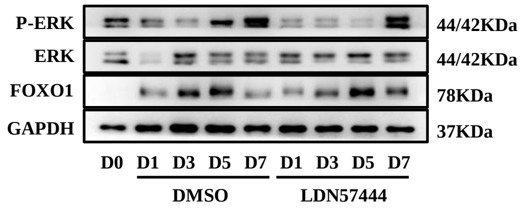

Supplementary Table 1. The sequences of primers

Name	Primer (5’ - 3’)
GAPDH	GGATTTGGTCGTATTGGG
	GGAAGATGGTGATGGGATT
UCHL1	CCTGTGGCACAATCGGACTTA
	CATCTACCCGACATTGGCCTT
IGFBP1	TTGGGACGCCATCAGTACCTA
	TTGGCTAAACTCTCTACGACTCT
PRL	TGACCCTTCGAGACCTGTTTG
	CTTGCTCCTTGTCTTCGGG
BMP2	ACCCGCTGTCTTCTAGCGT
	TTTCAGGCCGAACATGCTGAG
CCL2	CAGAAGTGGGTTCAGGATTCC
	ATTCTTGGGTTGTGGAGTGAG
IL-15	TTGGGAACCATAGATTTGTGCAG
	AGAGAAAGCACTTCATTGCTGTT
TGFβ1	CTAATGGTGGAAACCCACAACG
	TATCGCCAGGAATTGTTGCTG
CXCL9	GTGGTGTTCTTTTCCTCTTGGG
	ACAGCGACCCTTTCTCACTAC
CXCL10	GTGGCATTCAAGGAGTACCTC
	TGATGGCCTTCGATTCTGGATT
CXCL11	GACGCTGTCTTTGCATAGGC
	GGATTTAGGCATCGTTGTCCTTT
CXCL12	ATTCTCAACACTCCAAACTGTGC
	ACTTTAGCTTCGGGTCAATGC
IDO	GCCAGCTTCGAGAAAGAGTTG
	ATCCCAGAACTAGACGTGCAA
OPN	CTCCATTGACTCGAACGACTC
	CAGGTCTGCGAAACTTCTTAGAT
Uchl1	GATGCTGAACAAAGTGTTGGC
	GGAGTTTCCGATGGTCTGCTT
Igfbp1	CTGCCAAACTGCAACAAGAATG
	GGTCCCCTCTAGTCTCCAGA
Prl	TTCTGCCAAAATGTTCAGCCT
	CTTGACCATAAACTCACGGTCTT
Actb	GGCTGTATTCCCCTCCATCG
	CCAGTTGGTAACAATGCCATGT

Supplementary Table 2. Clinical characteristics of patients with unexplained spontaneous abortions and clinically normal pregnancy involved in this study.

Characteristics	Unexplained spontaneous abortion patients (n=20)	Normal pregnant patients (n=20)
Age (years)	31.5±1.2	30.9±2.7
BMI (kg/m^2)	22.5±2.8	21.7±2.1
Gestational age (weeks)	8.3±4.2	7.8±2.5
Number of miscarriages	1.8±1.5	None
Duration of infertility (years)	0	0
Irregular menstrual cycles (n, %)	6, 17.6%	6, 16.7%
